# Hydrogen gas alleviates lipopolysaccharide-induced acute lung injury and inflammatory response in mice

**DOI:** 10.1186/s12950-022-00314-x

**Published:** 2022-10-17

**Authors:** Hongling Yin, Yajing Feng, Yi Duan, Shaolin Ma, Zhongliang Guo, Youzhen Wei

**Affiliations:** 1 Research Center for Translational Medicine & Key Laboratory of Arrhythmias of the Ministry of Education of China, East Hospital, Tongji University School of Medicine, 150 Jimo Road, Shanghai, 200120 China; 2 Department of Center ICU, East Hospital, Tongji University School of Medicine, 150 Jimo Road, Shanghai, 200120 China; 3 Department of Critical Care Medicine, East Hospital, Tongji University School of Medicine, 150 Jimo Road, Shanghai, 200120 China; 4 Department of Respiratory Medicine, Shanghai East Hospital, Tongji University School of Medicine, 150 Jimo Road, Shanghai, 200120 China

**Keywords:** Lipopolysaccharide (LPS), Acute lung injury (ALI), Hydrogen (H_2_), Inflammation, Toll-like receptor 4(TLR4), Nuclear factor kappa-B (NF-κB)

## Abstract

**Background:**

Chronic inflammation and oxidant/antioxidant imbalance are two main pathological features associated with lipopolysaccharide (LPS)-induced acute lung injury (ALI). The following study investigated the protective role of hydrogen (H_2_), a gaseous molecule without known toxicity, in LPS-induced lung injury in mice and explored its potential molecular mechanisms.

**Methods:**

Mice were randomly divided into three groups: H_2_ control group, LPS group, and LPS + H_2_ group. The mice were euthanized at the indicated time points, and the specimens were collected. The 72 h survival rates, cytokines contents, pathological changes, expression of Toll-like receptor 4 (TLR4), and oxidative stress indicators were analyzed. Moreover, under different culture conditions, RAW 264.7 mouse macrophages were used to investigate the potential molecular mechanisms of H_2_ in vitro. Cells were divided into the following groups: PBS group, LPS group, and LPS + H_2_ group. The cell viability, intracellular ROS, cytokines, and expression of TLR4 and nuclear factor kappa-B (NF-κB) were observed.

**Results:**

Hydrogen inhalation increased the survival rate to 80%, reduced LPS-induced lung damage, and decreased inflammatory cytokine release in LPS mice. Besides, H_2_ showed remarked anti-oxidative activity to reduce the MDA and NO contents in the lung. In vitro data further indicated that H_2_ down-regulates the levels of ROS, NO, TNF-α, IL-6, and IL-1β in LPS-stimulated macrophages and inhibits the expression of TLR4 and the activation of nuclear factor kappa-B (NF-κB).

**Conclusion:**

Hydrogen gas alleviates lipopolysaccharide-induced acute lung injury and inflammatory response most probably through the TLR4-NF-κB pathway.

**Supplementary Information:**

The online version contains supplementary material available at 10.1186/s12950-022-00314-x.

## Introduction

Acute lung injury (ALI) is a severe disease syndrome with high morbidity and high mortality [1–3]. Its pathogenesis is complex and characterized by inflammatory processes, oxidative stress, apoptosis, and increased autophagy [4]. Treatment of ALI is based on both ventilatory and nonventilatory strategies. Yet, the efficacy of standard therapies such as prone positioning and administration of neuromuscular blocking agents, glucocorticoids and surfactants stem cells injection, and lung-protective mechanical ventilation is limited [5–7]. Thus, there is an urgent need to develop novel therapeutic strategies and agents for ALI.

Lipopolysaccharide (LPS) is an outer membrane of Gram-negative bacteria and a common inducing agent of ALI [8]. LPS activates the macrophages and inflammatory cells, which then release uncontrolled inflammatory cytokines [9]. Studies have suggested that LPS-activated Toll-like receptors 4 (TLR4) can generate inflammatory cytokines by activating the NF-κB signaling pathway [10–13]. These cytokines exert critical roles in ALI development by amplifying inflammatory responses [3]. Thus, targeted inhibition of TLR4 and downstream NF-κB signaling pathways, breaking the cascade of inflammation, may be a promising strategy for ALI.

Inflammatory lung diseases are characterized by inflammation development and oxidant/antioxidant imbalance. The balance between the production and elimination of ROS maintains the functional integrity of redox-sensitive signaling [14]. ROS-activated redox-sensitive transcription factors, such as NF-κB, which regulate the chromatin remodeling and gene expression of pro-inflammatory mediators, can enhance inflammatory responses and tissue injury [15–17]. In this study, we hypothesized that inhibition of the downstream NF-κB signaling pathway might be a useful strategy for scavenging ROS and treating ALI.

Hydrogen (H_2_) is a gaseous molecule without known toxicity, which reacts with hydroxyl radical to remove the reactive oxygen species (ROS). Recent studies have revealed that hydrogen is an important physiological regulator with antioxidant, anti-inflammatory, and anti-apoptotic effects on cells and organs [18–21]. Moreover, studies showed that hydrogen in the human body acts as a regulator of signal transduction like other gaseous signaling molecules and thus has been proposed as ‘the fourth signal gas molecule’ [22, 23]. However, whether H_2_ improves the specific anti-inflammatory mechanism of LPS-induced ALI is still unclear.

This study aimed to investigate the protective role of H_2_ in LPS-induced lung injury and explore its potential molecular mechanisms.

## Material and methods

### In vivo experimental design

#### Animals and experimental design

A total of 105 female C57BL/6 (6–8 weeks of age and weighing 19–22 g) mice were assessed in the present study. All the animals were housed in an environment with a temperature of 22 ± 1 ºC, relative humidity of 50 ± 1%, and a light/dark cycle of 12/12 h and were given water and food ad libitum. All animal studies (including the mice euthanasia procedure) were done in compliance with the regulations and guidelines of Tongji University School of Medicine in Shanghai institutional animal care and conducted according to the AAALAC and the IACUC guidelines (Shanghai) 2017–0005).

All mice were randomly divided into 3 groups (*n* = 35): 42% hydrogen gas inhalation group (H_2_ group), LPS-induced ALI group (LPS group, LPS derived from *Escherichia coli* (serotype O111:B4)), and ALI with 42% hydrogen inhalation group (LPS + H_2_ group).

Ten mice were randomly drawn from each group to determine the 72 h survival statistics, which were used to assess the protective effect of H_2._ The other 75 mice were used for collecting other samples. The two ALI groups were induced by intraperitoneal injection of 10 mg/kg LPS. Treatments with inhalation of 42% hydrogen gas for 72 h were administered after the injection of LPS or saline. Hydrogen was produced by a hydrogen–oxygen nebulizer (license No: AMS-H-03, Shanghai Asclepius Meditec Co., Ltd., Shanghai, China) that generates 3 L/min hydrogen gas by water electrolysis. As measured by gas chromatography, the gas generated consisted of 67% hydrogen and 33% oxygen. To keep the oxygen content at 21%, a certain amount of nitrogen was passed in. Thus, the hydrogen mixed gas contained 42% hydrogen, 21% oxygen, and 37% nitrogen (endotoxin-free, purity > 99.9%) in the air.

#### Cytokine measurements

To measure the cytokines in the serum of mice, blood samples were collected from the eyeball at 3, 6, 12, 24, 48, and 72 h after LPS injection. The serum was separated by centrifugation at 3000 g for 15 min (4℃) and then stored at -80℃. The corresponding enzyme-linked immunosorbent kit (CUSABIO BioTECH., Ltd., China) was used to detect the concentrations of IL-1β, TNF-α, IL-6, and IL-10. All samples were measured in triplicate.

#### Measurement of MDA and NO levels in lung tissues

Malondialdehyde (MDA) and nitric oxide (NO) are commonly used to represent local or systemic oxidative stress. The lung tissues were collected at 6, 12, and 24 h after LPS injection. Then, 10% of lung tissue homogenates were separated from the supernatant by 3000 g centrifugation for 20 min at 4 °C. Subsequently, MDA and NO content in the supernatants was measured using an MDA assay kit and NO assay kit (Jiancheng Bioengineering Institute, Nanjing, China).

#### Histological examinations

Lung tissue was collected at 6, 12, 24, and 48 h after LPS injection and fixed in 10% neutral buffered formalin for 24 h and then embedded in paraffin. All specimens were cut into 5 μm thick sections and stained with hematoxylin and eosin (HE). Samples were photographed and examined immediately by Leica DM Microscopes (DM 2500B, Germany, × 200).

#### TLR4 expressions in lung tissues

Twelve and 24 h after LPS injection, lung specimens were cut into 5 μm thick sections, then immersed in 3% H_2_O_2_ for 25 min and rinsed with PBS. They were blocked with 10% normal rabbit serum for 30 min at room temperature and stained with anti-TLR4 primary antibody (1:500; Abcam) overnight at 4 °C, followed by horseradish peroxidase or fluorescein isothiocyanate-conjugated goat anti-mouse immunoglobulin G antibody (1:5000; zhongshan bio.) for 50 min at room temperature. Sections were observed under a microscope (Eclipse Ti-SR; Nikon, Japan, × 200).

### In vitro experimental design

#### Cell Culture and groups

The mouse RAW 264.7 macrophage cells were purchased from American Type Culture Collection [ATCC], USA. Cells were cultured in a humidified incubator containing 5% CO_2_ with Dulbecco’s modified Eagle’s medium (DMEM) medium (Gibco, Grand Island, NY) at 37 °C, containing 100-IU/mL penicillin G, 100-IU/ mL streptomycin, and 10% heat-inactivated fetal bovine serum.

The experiment included three groups: PBS group, LPS group, and LPS + H_2_ group. The PBS group was cultured with a normal DMEM medium and stimulated with PBS in a humidified atmosphere containing 5%CO_2_/95% air at 37ºC; the LPS group was cultured with a normal DMEM medium and stimulated with LPS in a humidified atmosphere containing 5%CO_2_/95% air at 37ºC; the LPS + H_2_ group was cultured with normal DMEM medium + 60% H_2_ and stimulated with LPS in a humidified atmosphere containing hydrogen mixed gas (60% hydrogen, 21% oxygen, 5% carbon dioxide, and 14% nitrogen (endotoxin-free, purity > 99.9%) in air. Hydrogen was produced by a hydrogen–oxygen nebulizer (license No: AMS-H-03, Shanghai Asclepius Meditec Co., Ltd., Shanghai, China).

#### Nitric oxide measurement

RAW 264.7 mouse macrophages (1 × 10^5^cell/ml) were plated in 96-well plates. On day 2, the LPS + H_2_ group and LPS group were pretreated with H_2_ (60% H_2_ + normal cell medium) or vehicle (normal cell medium) for 24 h, respectively, and then all cells were incubated with LPS (200 ng/mL) for another 24 h. The concentration of nitric oxide (NO) in media was determined using the NO assay kit (Jiancheng Bioengineering Institute, Nanjing, China).

#### Cell viability assay

Cell viability was assessed by the Cell Counting Kit (Beyotime, China). In 96-well culture dishes, 1 × 10^5^ macrophages in each well and cell were pretreated with 60% H_2_ for 24 h and then stimulated with LPS (200 ng/ml) or PBS for another 24 h. Then, a 20 μl CCK-8 solution was added to each well and incubated at 37 °C for another 4 h. The cell viability was measured by a microplate spectrophotometer (Thermo, USA) at OD450 nm.

#### Detection of intracellular ROS

Intracellular ROS production was monitored by a ROS assay kit (Solarbio, China). RAW 264.7 mouse macrophages (1 × 10^5^cell/ml) were seeded into 6-well. Cells were pretreated with 60% H_2_ for 24 h and then stimulated with LPS (1 μg/ml) or PBS for another 24 h. Then, cells were exposed to a serum-free medium containing 10-μM 2', 7'-Dichlorodihydrofluorescein diacetate (DCFDA). After 20 min of incubation in darkness, the cells were washed three times with a blank DMEM medium. At last, cells were observed under the fluorescence microscope (OLYMPUS, IX71).

#### Real-time quantitative PCR

In 6-well culture dishes, 1 × 10^5^ macrophages were placed in each well and pretreated with 60% H_2_ for 24 h and then stimulated with LPS (1 μg/ml) or PBS for another 24 h. Total RNA was extracted by Trizol reagent (Invitrogen, USA) and reverse-transcribed using Thermo Scientific RevertAid cDNA Synthesis Kit (Thermo, USA) to produce cDNA. The quantitative real-time PCR was performed with the ABI-7500 machine using SYBR Green PCR Kit (Thermo, USA).Glyceraldehydes-3-phosphate dehydrogenase (GAPDH) mRNA was used as an internal control. The quantitative PCR program used was as follows: predenaturation (94 °C, 10 min), denaturation (94 °C, 20 s), annealing (55 °C, 20 s), and extension (72 °C, 20 s), using primers specific for GAPDH, IL-6, IL‐1β, IL‐10, and TNF-α.

Each sample was conducted in triplicate, and the gene expression levels were calculated relative to the amount of GAPDH using the 2^−ΔΔCT^ method. The primer sequences for the tested genes are listed in Table 1.

**Table 1:** The primer sequences of mouse GAPDH, TNF-α, IL-1β, IL-6 and IL-10

Gene	Sequences
GAPDH-F	AGGTCGGTGTGAACGGATTTC
GAPDH-R	TGTAGACCATGTAGTTGAGGTCA
TNF-α-F	GAGTGACAAGCCTGTAGCC
TNF-α-R	CTCCTGGTATGAGATAGCAAA
IL-1β-F	GATCCACACTCTCCAGCTGCA
IL-1β-R	CAACCAACAAGTGATATTCTCCAT
IL-6-F	AGTCCGGAGAGGAGACTTCA
IL-6-R	ATTTCCACGATTTCCCAGAG
IL-10-F	AGCCGGGAAGACAATAACTG
IL-10-R	CATTTCCGATAAGGCTTGG

### Western blot

All cells were collected and lysed in RIPA buffer (Beyotime, China), and the whole proteins, nucleoproteins, and cytoplasmic proteins were extracted as required. Protein concentrations were determined by BCA protein assay kit (KeyGEN BioTECH, China). Equal quantities of protein were separated on 12% SDS–PAGE, electrophoretically transferred to polyvinylidene fluoride membranes (Millipore, USA), and then blocked with 5% non-fat milk in TBST buffer for 2 h at room temperature. The membranes were then incubated with the corresponding antibodies overnight at 4 °C. The corresponding antibodies were: anti-IκB, anti-pIκB, anti-TLR4, and anti-NF-κB(Abcam, Cambridge, UK). Samples were then washed three times and incubated with the horseradish peroxidase-conjugated secondary anti-rabbit/mouse antibody for 1 h at room temperature. The proteins were visualized using an enhanced ECL detection kit (Dingguo changsheng biotechnology CO., Ltd., China) and scanned with a Clinx ChemiScope chemiluminescence imaging system (ChemiScope 5300 Pro). The relative optical densities of specific proteins were estimated utilizing a ChemiScope analysis program.

### Statistical analysis

Data were reported as the mean ± SEM. All statistical analyses were performed using Prism 5.0 (GraphPad Software, USA). The nonparametric Mann–Whitney U test was employed to compare the value of all indicators between pairwise. A significance level of 0.05 was considered to be significant for all calculations. #p or *p represents *p*-value < 0.05. ##p or **p represents *p*-value < 0.01. ###p or ***p represents *p*-value < 0.001.

## Result

### Hydrogen inhalation improved the survival rate of ALI-mice

**Fig. 1:**
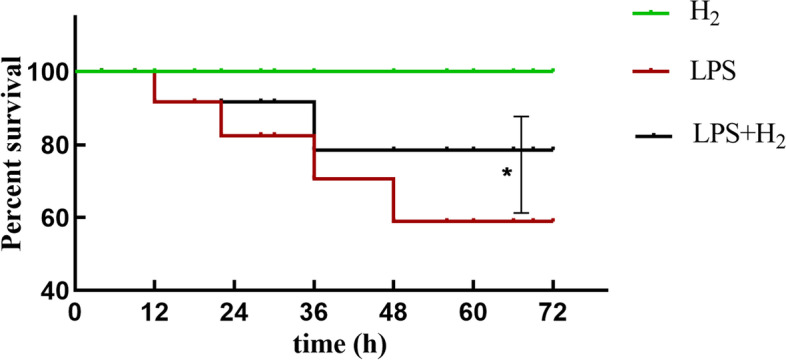
Effects of H_2_ on the survival rate of mice. Results are expressed as percent survival, *n* = 10. *P < 0.05 values of LPS mice compared with the LPS + H_2_ mice

The 72 h survival rates were recorded after experiments and analyzed by Log-rank (Mantel-Cox) test. As shown in Fig. 1A, the survival of mice in the H_2_ group was 100%, 60% in the LPS group, and 80% in the H_2_ treatment group (all *p* < 0.05).

### Hydrogen inhalation reduced the oxidative stress of ALI-mice

**Fig. 2:**
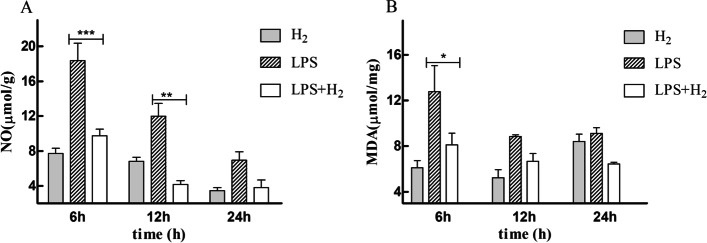
Effects of H_2_ on MDA levels and NO content. H2 treatment significantly reduced the increased NO and MDA levels induced by LPS. (A) NO levels; (B) MDA levels. *P < 0.05 and **P < 0.01 values of LPS mice compared with the LPS + H2 mice

MDA and NO concentration was an index of lipid peroxidation. The levels of oxidative product (NO and MDA) in lung tissues were measured at 6, 12, and 24 h after LPS administration. As shown in Fig. 2, NO and MDA were significantly decreased in mice treated with H_2_ (all *p* < 0.01).

### Hydrogen inhalation ameliorated inflammatory responses of ALI-mice

**Fig. 3:**
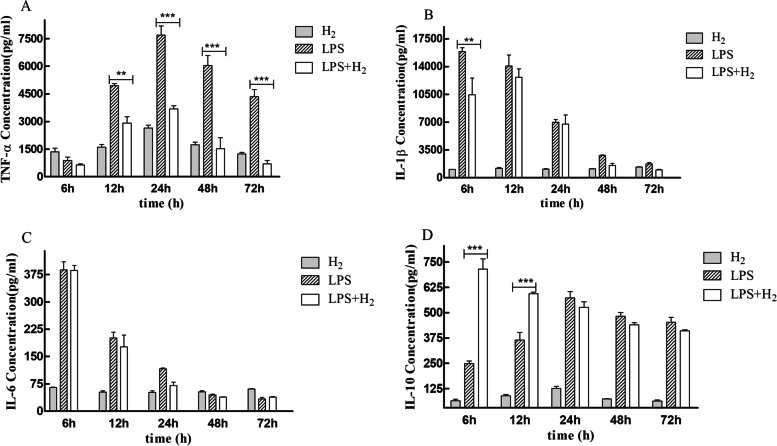
Effect of H_2_ on inflammatory cytokines in the serum of mice after LPS stimulation. (A) TNF-a; (B) IL-1β; (C) IL-6; (D) IL-10. *P < 0.05, **P < 0.01 and ***P < 0.001 values of LPS mice compared with the LPS + H_2_ mice

**Fig. 4:**
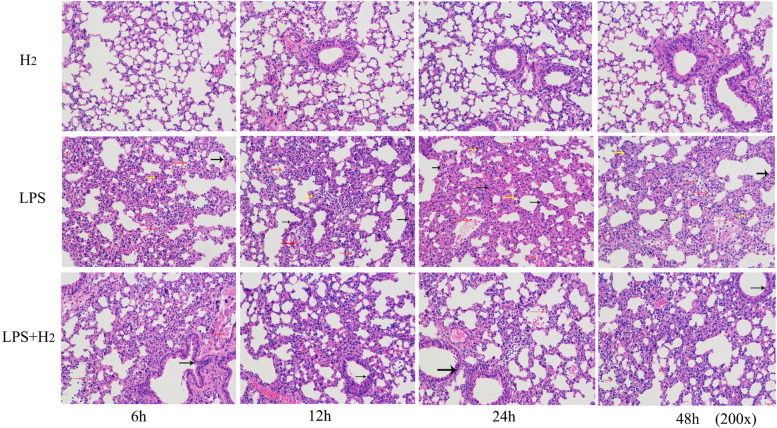
Effects of H_2_ treatment on histological changes in lung of LPS-induced ALI mice (× 200)

Cytokines that promote inflammation like TNF-α, IL-1β, IL-6, and adjusted inflammation like IL-10 were chosen to represent the systematic inflammation level. Figure 3A, B, and C represent TNF-α, IL-1β, and IL-6 levels in serum, respectively. The results showed the LPS induced TNF-α and IL-1β; contrary, these expressions were markedly reduced in the H_2_ treatment group. However, the level of IL-6 did not change after treatment. Furthermore, Fig. 4D showed that IL-10 level was significantly higher than the control group in a short time (< 24 h).

### Hydrogen inhalation reduced the lung injury of ALI-mice

Histological observation using H&E stain is shown in Fig. 4. Alveolar collapse, alveolar septal thickening, and a inflammatory cell infiltration were obvious in the lung tissue of LPS mice (Marked with yellow, black and red arrows respectively). This histological examination showed that LPS induced oxidative stress and inflammations, which resulted in tissue injury, and was consistent with the lipid peroxidation and cytokines results. Contrary, H_2_ inhalation significantly prevented the histopathological changes caused by LPS.

### Hydrogen inhalation reduced the expression of TLR4

**Fig. 5:**
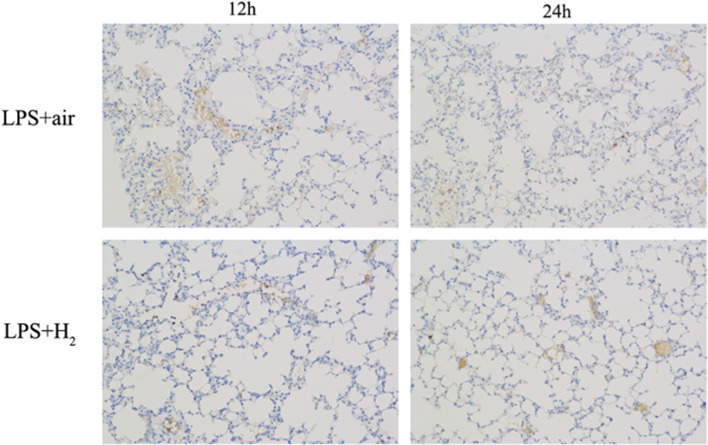
The expression of TLR4 in mice lung tissues analyzed by immunohistochemical staining in the LPS and LPS + H_2_ groups (× 200)

Immunohistochemistry was performed to explore the effects of H_2_ on the expression of TLR4 in lung tissues. As shown in Fig. 5, H_2_-treated mice showed lower TLR4 expression compared to LPS mice.

### Hydrogen reduced the release of the cytokine in LPS-stimulated macrophages

**Fig. 6:**
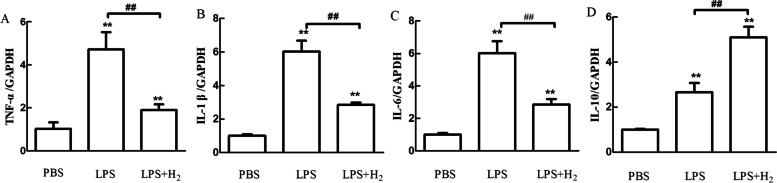
Effect of H_2_ (pretreated with H_2_ for 24 h) on inflammatory cytokines. (A) TNF-a; (B) IL-1β; (C) IL-6; (D) IL-10. **P < 0.01values of LPS + H_2_ and LPS group compared with the PBS group. ##*P* < 0.01values of LPS + H_2_ group compared with the LPS group

To assess the inhibitory role of H_2_ on cytokine production, we used the quantitative real-time PCR assay to detect the TNF-α, IL-6, IL-1β, and IL-10 mRNA expression in macrophages after LPS stimulation. Compared with the basal level without LPS stimulation, the contents of the four cytokines were significantly increased. At the same time, the TNF-α, IL-6, and IL-1β mRNA levels were all inhibited by H_2_ (Fig. 6). These findings indicate that H_2_ could neutralize the pro-inflammatory mediator effect of LPS, showing an anti-inflammatory effect.

### Hydrogen increased cell viability and reduced the NO level in RAW 264.7 Cells

**Fig. 7:**
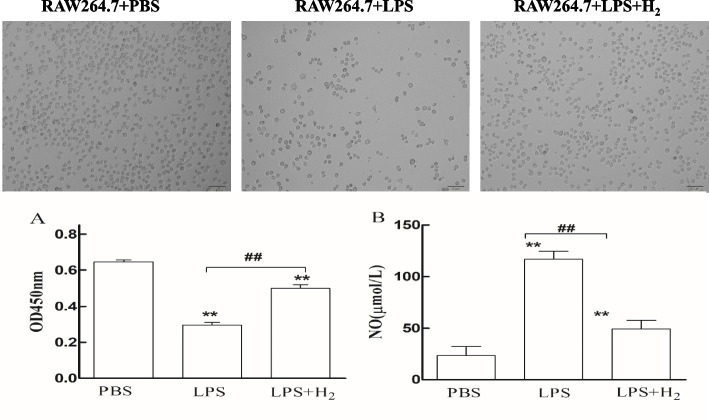
Cell viability and NO level of different groups (pretreated with H_2_ for 24 h). (A) Cell viability; (B) NO levels. **P < 0.01values of LPS + H_2_ and LPS group compared with the PBS group. ##*P* < 0.01values of LPS + H_2_ group compared with the LPS group

After LPS stimulation, cell proliferation was significantly reduced compared with the PBS and LPS + H_2_ groups (Fig. 7). Also, the NO content increased significantly (116.632 ± 7.729 μmol/L) compared to PBS (23.464 ± 8.615 μmol/L) and LPS + H_2_ groups (48.999 ± 8.661 μmol/L).

### Hydrogen reduced the ROS production in LPS-stimulated RAW 264.7 cells

**Fig. 8:**
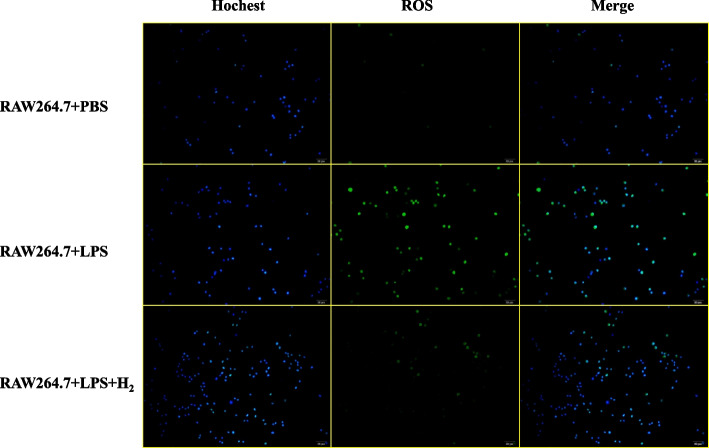
The ROS level of RAW 264.7 + PBS group, RAW 264.7 + LPS group, and RAW 264.7 + LPS + H_2_ group (pretreated with H_2_ for 24 h)

The RAW 264.7 + PBS group showed good cell status and low intracellular ROS levels. After LPS stimulation, the cell status deteriorated, and ROS levels increased significantly. When H_2_ treatment was performed before LPS stimulation, intracellular ROS levels were significantly reduced (Fig. 8).

### Hydrogen reduced the TLR4 expression and suppressed NF-κB activation in RAW 264.7 cells caused by LPS

**Fig. 9:**
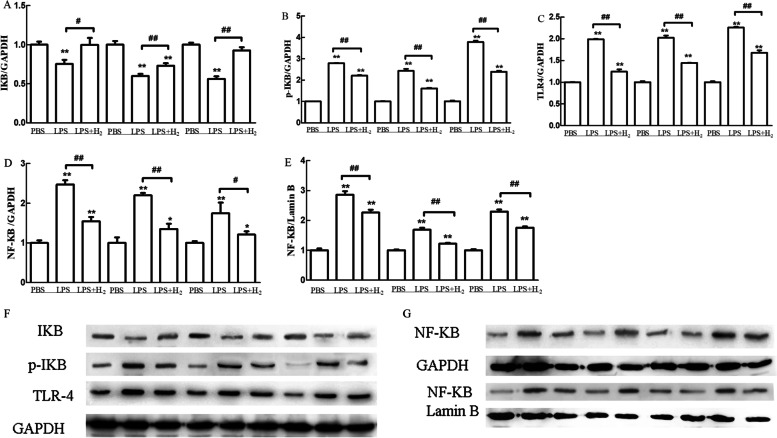
H_2_ inhibits the TLR4 expression and NF-κB activation (pretreated with H_2_ for 24 h). Expression of GAPDH (Proteintech, USA) and Lamin B (Abcam, UK) were internal controls. The values presented are the means ± SEM of three independent experiments. ##*p* < 0.01 compared to PBS group; *p < 0.05 and **p < 0.01 compared to LPS group

Compared with the control group, the expression level of IκB was significantly decreased after stimulation with LPS at 1 μg/ml, while the expression levels of phosphorylated IκB, TLR4, and NF-κB (cytoplasm and nucleus) were significantly increased (Fig. 9). After 60% H_2_ treatment, the expression level of IκB was significantly increased, while the phosphorylated IκB, TLR4, and NF-κB (cytoplasmic and nuclear) expression levels were significantly reduced compared to the LPS stimulation group.

## Discussion

Acute lung injury (ALI) is a serious inflammatory disease [1–3]. LPS is a key mediator of organ dysfunction and death associated with severe gram-negative infections [8] and the most important pathogen causing ALI [24, 25]. In this study, we used LPS (i.p., 10 mg/kg) to establish the ALI model mice.

Recent studies have shown that hydrogen gas (H_2_) is an important physiological regulatory factor with antioxidant, anti-inflammatory, and anti-apoptotic properties [26]. In the present study, we investigated the protective effects of H_2_ treatment in LPS-induced ALI mice. We hypothesized that its protective effect might be related to its ability to ameliorate the extent of oxidative stress and prevent the release of pro-inflammatory molecules [14–17]. H_2_ inhalation can reduce lung injury caused by the ventilator, transplantation, hyperoxia, irradiation, and sepsis [27–30]. The histopathologic evaluation shows that the lung in the LPS group showed a thickened alveolar wall, edema and hemorrhage, less alveolar space, and inflammatory cell infiltration after LPS stimulation, which, however, were not observed in LPS mice treated with H_2_ inhalation. The H_2_ treatment ameliorates LPS-induced lung neutrophil infiltration and inflammation.

To verify the success of the ALI model, we observed the pathological and inflammatory changes at different time points after LPS injection in mice. The critical feature of ALI is the lung parenchyma injury and acute inflammatory process, including the release of inflammatory mediators such as TNF-α, IL-1β, and IL-6 [31]. Pro-inflammatory cytokines appear in the early stages of inflammation, indicating the severity of ALI in a certain sense [32]. In this study, the inflammatory factors gradually increased during the first 24 h after LPS administration compared to the H_2_. These results suggested that the ALI model was successfully established after intraperitoneal injections of LPS, with the lung injury being obvious at 24 h point. H_2_ markedly reduced the inflammation of ALI.

In vivo data further indicated that hydrogen gas inhalation protects mice against ALI lethality. It markedly reduces the inflammation and oxidative damages caused by ALI, improves the lung injury caused by LPS stimulation, and inhibits the TLR4 expression in lung tissues. In conclusion, these results show that molecular hydrogen alleviates LPS-induced ALI by reducing lung inflammation and oxidative damage, which may be associated with decreased NF-κB activity. All of the results are consistent with Sun et al. [33] and Du et al. [34], who confirmed that hydrogen could alleviate LPS-induced pulmonary lesions and pulmonary edema and reduce the degree of ALI by inhibiting the release of pro-inflammatory cytokines and oxidative stress. However, Sun’s study results further discussed the apoptosis-related results and the effect of the *Nrf2* gene on the NF-KB pathway, while Du et al.explained the effect of the *sirt1* gene on the NF-KB pathway.

LPS causes decrements in neutrophilic inflammation and pulmonary function via Toll-like receptor 4 to induce the expression of inflammatory cytokines and chemokines [35, 36]. The mechanism of ALI injury may be associated with bacterial endotoxin (LPS) activation that promotes the interaction of TLR4 and results in activation of NF-κB and release of TNF-α and IL-6 [37]. All of these factors have a pivotal part in inflammatory lung damage. Many studies have proved that inhibiting the activity of NF-κB in different models can alleviate the tissue damage and down-regulated release of cytokines [38, 39]. Therefore, inhibition of NF-κB activation may be an effective choice for protecting ALI.

H_2_ can directly activate the NF-κB signaling by inhibiting the phosphorylation of IκB-α [40]. In addition, H_2_ can inhibit the activation of the NF-κB signaling pathway by scavenging the oxygen radicals [41]. Hydrogen gas, a potent antioxidant with rapid gaseous diffusion, effectively reduces cytotoxic free radicals, such as reactive oxygen species (ROS), whilst being mild enough to cause interference with metabolic redox reactions or disrupt cell signaling. Our in vitro data further showed that the elevations of TNF-α, IL-6, and IL-1β mRNA levels were suppressed by H_2_. Moreover, we found that H_2_ alleviates the cell damage induced by LPS and reduces the ROS and NO levels of the cell. In addition, the intracellular ROS levels were significantly reduced in the LPS + 60%H_2_ group, and H_2_ inhibited TLR4 expression and NF-κB activation in macrophages caused by LPS.

In this study, LPS administration induced massive inflammatory cells and the release of cytokines in the serum, which were attenuated by H_2_ inhalation treatment. Moreover, we assessed the lung oxidative damage by measuring the level of MDA and NO, and the results showed that hydrogen gas decreased the oxidative lung damage caused by LPS. On the other hand, H_2_ dramatically inhibited the release of pro-inflammatory cytokines (TNF-α and IL-1β) in LPS-challenged mice and significantly up-regulated anti-inflammatory cytokines (IL-10). Previous studies reported that H_2_ inhalation could inhibit the release of pro-inflammatory cytokines [42]; however, there are no studies about its effect on anti-inflammatory cytokines.

## Conclusions

In vivo and in vitro studies demonstrated that inhalation of H_2_ could relieve LPS-induced ALI and downregulate the TLR4-mediated NF-κB signaling pathway, thus inhibiting inflammation.

## Supplementary Information

**Additional file 1.** 

## Data Availability

All data generated or analyzed during this study are included in this published article.
