# Supplementary material for: Hydrogen gas alleviates lipopolysaccharide-induced acute lung injury and inflammatory response in mice
Source: J Inflamm (Lond). 2022 Oct 17;19:16. doi: 10.1186/s12950-022-00314-x (PMC9575233; doi:10.1186/s12950-022-00314-x)
Supplement: Supplementary file 1 — Additional file 1. [file 12950_2022_314_MOESM1_ESM.zip › Table 2.DOCX]

Table 1

The primer sequences of mouse GAPDH, TNF-α, IL-1β, IL-6 and IL-10

| Gene | Sequences |
| --- | --- |
| GAPDH-F | AGGTCGGTGTGAACGGATTTC |
| GAPDH-R | TGTAGACCATGTAGTTGAGGTCA |
| TNF-α-F | GAGTGACAAGCCTGTAGCC |
| TNF-α-R | CTCCTGGTATGAGATAGCAAA |
| IL-1β-F | GATCCACACTCTCCAGCTGCA |
| IL-1β-R | CAACCAACAAGTGATATTCTCCAT |
| IL-6-F | AGTCCGGAGAGGAGACTTCA |
| IL-6-R | ATTTCCACGATTTCCCAGAG |
| IL-10-F | AGCCGGGAAGACAATAACTG |
| IL-10-R | CATTTCCGATAAGGCTTGG |
