# Supplementary material for: Hydrogen gas alleviates lipopolysaccharide-induced acute lung injury and inflammatory response in mice
Source: J Inflamm (Lond). 2022 Oct 17;19:16. doi: 10.1186/s12950-022-00314-x (PMC9575233; doi:10.1186/s12950-022-00314-x)
Supplement: Supplementary file 1 — Additional file 1. [file 12950_2022_314_MOESM1_ESM.zip › Table 1.DOC]

**Research highlights:**

- Hydrogen inhalation protected mice against ALI lethality
- Hydrogen inhalation improved the lung injury caused by LPS stimulation significantly
- Hydrogen markedly reduced the inflammation and oxidative damages in ALI mice and macrophages
- Hydrogen inhibited the TLR4 expression, NF-κB activation in lung tissues and macrophages
